# Innovative techniques using a novel thin scope for stent placement in malignant colonic obstruction with severe angulation deformity

**DOI:** 10.1055/a-2418-3025

**Published:** 2024-10-14

**Authors:** Yosei Sawai, Tomohiro Shimada, Taku Yamagata, Yoshihide Kanno, Takeshi Shimizu, Daichi Komabayashi, Kei Ito

**Affiliations:** 1 506803 Gastroenterology, Public Interest Incorporated Foundation Sendai City Medical Center, Sendai, Japan

Endoscopic placement of a metal stent was attempted in an 80-year-old man with bowel obstruction due to cancer of the rectosigmoid colon ( Fig. 1 ). Because of the severe angulation deformity of the tract, the lumen could not be identified using a standard scope (CF-HQ290ZI; Olympus, Tokyo, Japan). Therefore, we switched to an EG-840TP scope (Fujifilm, Tokyo, Japan) 1 ( Fig. 2 ), a novel thin scope of outer diameter 7.9 mm for gastrointestinal therapeutic procedures. This scope has similar specifications to conventional therapeutic scopes, including an accessory channel with a diameter of 3.2 mm and an adequate down-angle function, making it useful for interventions in confined spaces. Using this scope, a guidewire was easily advanced into the oral side of the colonic obstruction with a secure frontal view of the intracancer lumen. Moreover, the scope could be advanced beyond the cancer stricture without resistance. A Hanarostent (Boston Scientific Japan K.K., Tokyo, Japan) was partially deployed at the proximal colon side with endoscopic imaging, and was then fully opened using a within-the-scope channel (intra-scope channel) stent release technique 2 ( Video 1 , Fig. 3 ).

**Fig. 1:**
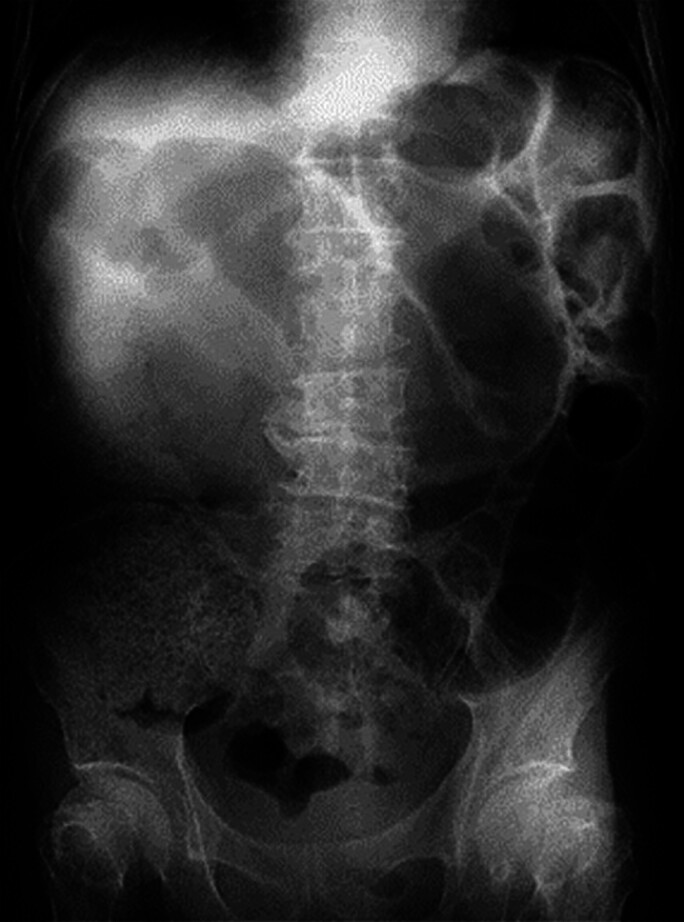
Abdominal radiographs in an 80-year-old man with bowel obstruction. Intestinal dilatation and niveau were observed.

**Fig. 2:**
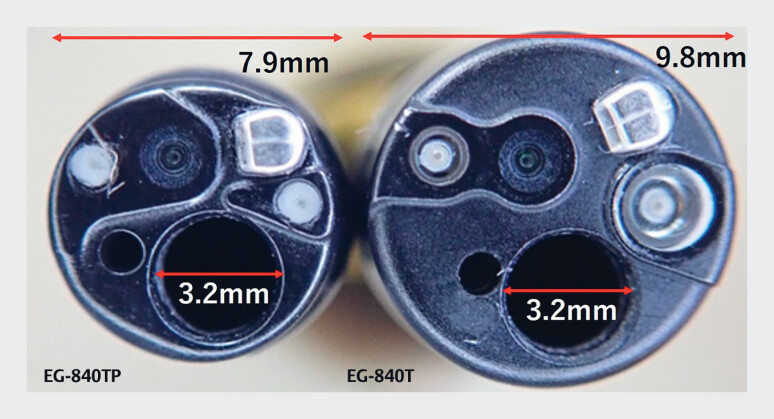
Comparison of the EG-840TP and EG-840T scopes. The EG-840TP is a novel thin scope with an outer diameter of 7.9 mm; it has similar specifications to conventional therapeutic scopes, including an accessory channel with a diameter of 3.2 mm.

**Fig. 3:**
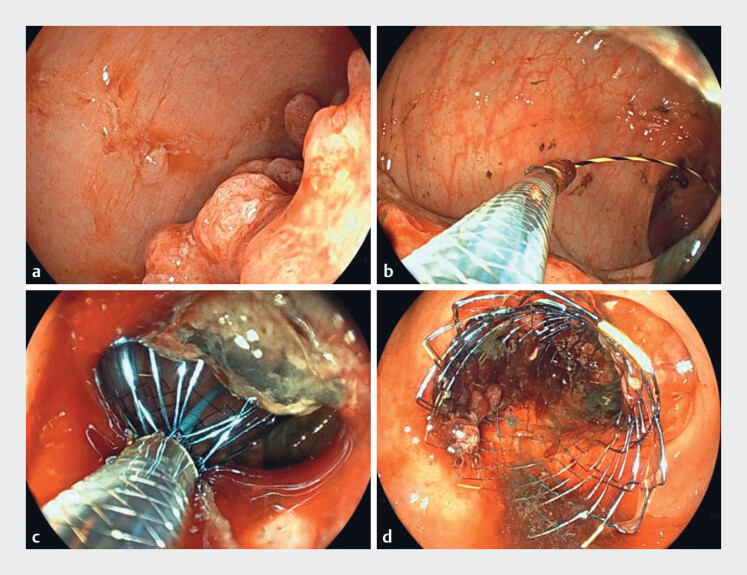
Stent placement using the novel thin scope. **a** The cancer is located at the severe angulation of the intestinal tract in the rectosigmoid colon. **b** A guidewire was easily advanced into the oral side of the obstruction; the scope could be advanced beyond the cancer stricture without resistance. **c** A stent was partially deployed at the proximal colon with endoscopic imaging, and stent placement was done using the intra-scope channel stent release technique. **d** The stent was placed in the appropriate position.

Stent placement in malignant colonic obstruction using a novel thin scope and the intra-scope channel stent release technique.Video 1

As the EG-840TP scope can be passed through severe strictures that are causing intestinal obstruction, its use might improve colorectal stent placement. Although the feasibility of this method needs to be evaluated using larger populations, it has the potential to simplify procedures and minimize X-ray exposure.

Endoscopy_UCTN_Code_TTT_1AQ_2AF

## Notes

Endoscopy E-Videos https://eref.thieme.de/e-videos
*E-Videos* is an open access online section of the journal *Endoscopy* , reporting on interesting cases and new techniques in gastroenterological endoscopy. All papers include a high-quality video and are published with a Creative Commons CC-BY license. Endoscopy E-Videos qualify for HINARI discounts and waivers and eligibility is automatically checked during the submission process. We grant 100% waivers to articles whose corresponding authors are based in Group A countries and 50% waivers to those who are based in Group B countries as classified by Research4Life (see: https://www.research4life.org/access/eligibility/ ). This section has its own submission website at https://mc.manuscriptcentral.com/e-videos .
